# Heparan sulfate in cerebrospinal fluid as a biomarker to assess disease severity and for treatment monitoring in patients with Mucopolysaccharidosis Type II: a position statement

**DOI:** 10.1186/s13023-024-03463-9

**Published:** 2024-11-26

**Authors:** Roberto Giugliani, Ana Cecília Menezes de Siqueira, Emerson Santana Santos, Emília Katiane E. A. Leão, Gerson da Silva Carvalho, Mara Lúcia Schmitz Ferreira Santos, Salmo Raskin, Ana Maria Martins

**Affiliations:** 1 Universidade Federal do Rio Grande do Sul, Hospital de Clínicas de Porto Alegre, Rua Ramiro Barcelos 2350, Porto Alegre, 90035-903 Brasil; 2 Dasa Genômica, Casa dos Raros, Porto Alegre, Brazil; 3 Instituto de Medicina Integral Professor Fernando Figueira, Recife, Brazil; 4 Universidade Federal de Sergipe, Lagarto, Brazil; 5 Hospital Universitário Professor Edgard Santos, Salvador, Brazil; 6 Centro de Referência em Doenças Raras, Hospital de Apoio de Brasília, Brasília, Brazil; 7 Hospital Pequeno Príncipe, Curitiba, Brazil; 8 Centro de Aconselhamento e Laboratório Genetika, Curitiba, Brazil; 9 Centro de Referência em Erros Inatos do Metabolismo, Universidade Federal de São Paulo, São Paulo, Brazil

**Keywords:** Biomarkers, Blood-brain barrier, Heparan sulfate, Cerebrospinal fluid, Mucopolysaccharidosis type II

## Abstract

Patients with mucopolysaccharidosis type II (MPS II) can present with a severe neuronopathic phenotype or an attenuated non-neuronopathic phenotype. In the light of the recent development of drugs that cross the blood-brain barrier for treatment of neurologic MPS II symptoms, it is critical to define biomarkers that objectively differentiate phenotypes and monitor therapeutic outcomes of advanced treatments. In December 2023, a panel of Brazilian experts discussed the potential of quantifying heparan sulfate (HS) in the cerebrospinal fluid (CSF) as a biomarker for assessing neurological impairment in patients with MPS II, as well as the potential of the molecule as an objective parameter for therapeutic monitoring. Based on scientific evidence, the experts concluded that HS in CSF is predominantly derived from the brain and reflects neurological impairment in patients with MPS II. CSF HS levels may help differentiate between neuronopathic and non-neuronopathic forms of MPS II, with preliminary observations suggesting a potential threshold around 4,000 ng/mL when HS quantification is performed using the same method described in clinical studies of pabinafusp alfa. According to the authors, monitoring HS levels in CSF can serve as an objective parameter for assessing the effectiveness of treatment with drugs that cross the blood-brain barrier. The recommended timing of HS evaluations in CSF of patients with the severe phenotype is: (i) before treatment; (ii) six months after starting treatment; and (iii) two years after starting treatment. The same monitoring scheme is recommended for patients with the attenuated MPS II phenotype, however, after two years of treatment, the physician may elect to perform regular neurocognitive evaluations instead of measuring HS in CSF. Lastly, the authors reinforced the importance of evaluating adherence to treatment, including interruptions, to provide a more meaningful assessment of the treatment’s real-world impact and to determine the ideal timing of CSF collection for therapeutic monitoring.

## Background

Mucopolysaccharidosis type II (MPS II), also known as Hunter Syndrome (OMIM #309900), is a genetic lysosomal storage disease, with an X-linked recessive inheritance, caused by deficiency of the enzyme iduronate-2-sulfatase. The deficiency in enzymatic activity, caused by pathogenic variants in the *IDS* gene, results in multisystemic accumulation of glycosaminoglycans (GAGs), mainly heparan sulfate (HS) and dermatan sulfate, which affects various cellular processes and activates different pathogenic cascades, leading to the development of a wide range of clinical manifestations [1, 2].

As in most MPS disorders, the signs and symptoms of MPS II include coarse facial features, hepatosplenomegaly, umbilical hernia, joint stiffness, obstruction of upper airways, and cardiac dysfunctions. In addition to the visceral and skeletal symptoms, many patients with MPS II present significant neurological impairment. Phenotypes are classified as severe (neuronopathic form, rapidly progressive) or attenuated (non-neuronopathic form, slowly progressive); it is estimated that approximately 2/3 of MPS II patients present the severe phenotype. Patients with the severe phenotype generally exhibit cognitive decline from the age of 3–4 years, while patients with the attenuated phenotype may present neurological alterations at more advanced stages of the disease, albeit less severe impairments than the marked cognitive decline seen in the severe form [1, 3].

Progressive deterioration of the central nervous system (CNS) is a feature in some MPS disorders, leading to neurobehavioral changes and cognitive decline, among other manifestations. Interestingly, CNS alterations are observed only in patients with MPS characterized by multisystemic accumulation of HS (MPS I, II, III, and VII) [3, 4].

HS is ubiquitously expressed and can be found on the cell surface, basal membrane, and extracellular matrix of all mammalian tissues. Besides being an important structural component, HS is also involved in the regulation of a number of cellular processes, such as cell migration, inflammatory response, receptor-mediated signaling, phagocytosis, morphogenesis, and organogenesis. In the CNS, HS plays a fundamental role in neuronal development as an essential component of the vascular basal membrane in the brain [5, 6].

Patients with MPS I, II, IIIA, IIIC, and IIID exhibit a higher level of HS in the cerebral cortex compared to age-matched controls, as observed in a *post-mortem* study [7]. Furthermore, evidence shows that the accumulation of HS is not restricted to lysosomes, with the molecule also found in various locations within the intra- and extracellular space in the CNS, impairing neurological function of patients with MPS I, II, III, and VII [8].

Therefore, determining the level of HS in brain tissue of MPS patients could provide relevant information for treatment, prognosis, and therapeutic monitoring. However, this assessment would require a highly invasive procedure (brain biopsy), rendering it unfeasible in clinical practice [9].

In MPS II animal models, a strong correlation was observed between the concentrations of HS in the brain and in cerebrospinal fluid (CSF), suggesting that HS levels in CSF could serve as a reliable indicator of HS concentrations in brain tissue [9, 10]. Similarly, studies have demonstrated that patients with MPS II have higher concentrations of HS in CSF compared to individuals without MPS [11, 12].

Additionally, HS levels in CSF are higher in MPS II patients with the severe phenotype than those with the attenuated phenotype, with a slight overlap. This suggests that HS level could serve as a predictor of disease severity, providing a more accurate prognosis [13].

Therefore, quantification of HS in CSF is a possible alternative for analyzing HS in the brain of patients with MPS II, representing an accessible means of investigating biochemical changes in the CNS, which is constantly exposed to the brain’s interstitial fluid [14]. The use of HS levels in CSF as a potential biomarker can offer a direct insight into the neurological status of MPS II patients and the effectiveness of treatments targeting the CNS.

Considering the need for additional tools assessing severity and monitoring treatment of patients with MPS II, a panel of eight Brazilian experts conducted an online meeting in December 2023 to discuss the available evidence in the literature and formulate a consensus on the potential of HS in CSF as a biomarker of severity in MPS II. This article reports the rigorous analysis carried out by the panel of experts and the consensus reached, with the aim of providing clear evidence-based recommendations to the medical community regarding the quantification of HS in cerebrospinal fluid as a biomarker in MPS II.

## Main text

This report was produced by a panel of eight recognized Brazilian experts in the field of mucopolysaccharidoses, with a specific focus on the evaluation of HS in CSF as a potential biomarker of disease severity in patients with MPS II. The panel meeting was held online in December 2023 according to a structured protocol that included the following steps:

(i) Literature review: before the meeting, a literature review of relevant scientific publications, clinical trials and experimental studies on MPS II and HS as a biomarker, was conducted. Articles that addressed the topic of the relationship between HS and the neurological manifestations of mucopolysaccharidoses were selected by the authors to provide a solid evidence base for subsequent discussions.
(ii) Question formulation: seven key questions were elaborated to guide the panel discussion. These questions were designed to cover different aspects of the use of HS in CSF as a biomarker of severity in MPS II and for therapeutic monitoring.
(iii) Discussion: during the online meeting, each question was presented and followed by an in-depth discussion. The panelists shared their perspectives based on the available literature, plus their extensive clinical and practical experience managing patients with MPS II, as well as in collection and analysis of CSF.
(iv) Statement formulation: after the discussion of each question, a consensus statement was formulated reflecting the panel’s position.
(v) Voting and consensus: the statements were put to a vote, with experts indicating their agreement or disagreement.

## Statements

All statements were formulated after a thorough discussion and reflect an unanimous position of the authors.

### HS in CSF of patients with MPS II is primarily derived from brain tissue

It was a consensus among experts that, based on evidence from animal models [9, 10], clinical studies [11, 13, 15] and *post-mortem* evaluation of brain tissue from MPS patients [7], HS found in CSF is primarily derived from brain tissue.

Correlation between brain HS and CSF HS has already been observed in MPS II mice. Specifically, the baseline concentrations of HS in the brain and their reduction after treatment with Iduronate-2-sulfatase fused with anti-hTfR antibody (pabinafusp alpha) were found to correlate with the levels in the CSF [9, 10].

A study with brain cortex tissues from *post-mortem* autopsy materials of patients with different types of MPS and age-matched controls showed that all patients with MPS II, except one patient with the attenuated phenotype, had significantly increased levels of brain HS [7]. In clinical trials, it has been demonstrated that patients with MPS II exhibit elevated levels of CSF HS, which significantly decrease following treatment with pabinafusp alpha [11, 13, 15].

The largest molecule documented to cross the blood-brain barrier, CINC1, measures approximately 7.8 kDa [16]; the molecular weight of HS is approximately 75 kDa, which prevents it from crossing the blood-brain barrier (or choroid plexus) and supports the hypothesis that HS in CSF originates from the CNS.

Therefore, experts agreed that HS levels in CSF serve as a reliable indicator of HS concentration in the brain of patients with MPS II.

### HS levels in CSF are a reliable biomarker of severity of neurological impairment in MPS II

As outlined above, the authors were in agreement that HS levels in CSF reflect the molecule’s concentration in the CNS. The fact that HS is involved in the pathophysiology of all MPS exhibiting neurological impairment [3, 4] was considered as strong evidence that the accumulation of the molecule in the CNS is associated with the neurological symptoms observed in MPS II.

Additionally, a clear difference between the levels of HS in the CSF of MPS II patients with severe compared to attenuated phenotypes, with little overlap, was demonstrated in a phase 2/3 trial of pabinafusp alfa (enzyme drug that crosses blood-brain barrier) [13].

Therefore, all authors agreed there is a strong correlation between concentration of HS in CSF and severity of neurological impairment in patients with MPS II.

### CSF HS levels may help differentiate between neuronopathic and non-neuronopathic forms of MPS II, with preliminary observations suggesting a potential threshold around 4,000 ng/mL when HS quantification is performed using the same method described in clinical studies of pabinafusp alfa

Determining the severity of neurological disease is essential for effective clinical and therapeutic management of MPS II patients. While initial observations suggest that CSF HS levels may help distinguish between neuronopathic and attenuated phenotypes of MPS II, the panel acknowledges that establishing definitive cutoff values would require dedicated validation studies that account for age-related variations, patients with intermediate phenotype, and other potential confounding factors. The panel concluded that further research is needed to establish evidence-based thresholds and to elucidate the scenario of patients with an intermediate phenotype. Moreover, it was emphasized that the cutoff value may vary depending on the methodology used to quantify HS levels, a factor which should be taken into account in data analysis [11, 13, 15, 17].

### Quantification of HS levels in CSF is an objective parameter for monitoring therapeutic effectiveness of drugs that cross the blood-brain barrier in patients with MPS II

Although detailed clinical evaluation and magnetic resonance imaging (MRI) are recognized as valuable tools in monitoring patients with MPS II, the emergence of new therapies capable of crossing the blood-brain barrier highlights the potential of HS levels in CSF as a biomarker with great utility in therapeutic monitoring.

A Phase 2 clinical trial investigating the efficacy of pabinafusp alfa demonstrated overall stabilization of neurocognitive function and adaptive behavior in patients with MPS II, regardless of disease severity. The favorable outcomes observed in neurocognitive assessments, as well as decrease in HS levels in the CSF strongly indicate successful delivery of the drug through the blood-brain barrier and a positive effect of the treatment on CNS pathology. The intensity of decrease of HS with intravenous therapies that bypass the blood-brain barrier is variable, and in some patients, this may be impacted by the formation of anti-drug antibodies [15].

A Phase 2/3 clinical trial was performed with intrathecal administration of idursulfase in patients with severe MPS II. There were trends towards a potential positive effect of idursulfase-IT across DAS-II composite, cluster, and subtest scores, notably in patients younger than 6 years at baseline. In a post hoc analysis, there was a significant (*p* = 0.0174), clinically meaningful difference in change from baseline in DAS-II GCA scores at week 52 with idursulfase-IT (*n* = 13) versus no idursulfase-IT (*n* = 6) among those younger than 6 years with missense iduronate-2-sulfatase gene variants. Overall, idursulfase-IT reduced cerebrospinal glycosaminoglycan levels from baseline by 72.0% at week 52. Idursulfase-IT was generally well tolerated. These data suggest potential benefits of idursulfase-IT in the treatment of cognitive impairment in some patients with neuronopathic MPS II [18].

Additionally, results from 5 years of intracerebroventricular idursulfase beta treatment showed a significant reduction of HS levels in the CSF, along with improvement of neurological symptoms in children with neuronopathic MPS II [19].

Altogether, these results were considered robust evidence supporting the use of HS levels in CSF as an objective parameter for therapeutic monitoring. The authors recognized the utility of this biomarker, given aspects such as: (i) the practical difficulty of performing MRI at many centers due to the lack of equipment or absence of trained professionals to analyze images in patients with MPS; (ii) limitations regarding the application of cognitive and behavioral tests, arising due to the disease itself or the absence of validated tests for MPS; and (iii) the impossibility of directly accessing the CNS to measure HS in the brain. In addition, CSF samples can be readily sent to laboratories for HS quantification.

### In patients with the severe phenotype of MPS II, HS levels in the CSF should be determined before the start of treatment, at six months, and at two years after treatment. In cases of treatment interruption or low adherence during the period, results should be evaluated with caution, or the timing of sample collection reviewed

The definition of the ideal timing of evaluation of HS in CSF was a topic of extensive debate, in which the experts considered various factors: (i) kinetics of HS reduction after start of treatment; (ii) individual variability in response to treatment; (iii) stability of HS levels during treatment; (iv) patient adherence to treatment and interruptions; and (v) logistics associated with the CSF collection procedure.

Based on clinical studies and the experience of each expert, the authors unanimously proposed that evaluations of HS should be carried out in regular clinical practice before starting treatment with drugs that cross the blood-brain barrier, at six months, and after two years of treatment. In the event of treatment interruption (> 3 weeks) or low adherence during the period (< 80%), the physician should analyze the results with caution or reschedule sample collection, as these factors can negatively impact reduction in HS.

To date, there are no data on HS levels in CSF after two years of treatment. Therefore, the authors believe that the need to perform the examination after this period or whether the patient can be followed clinically (especially if HS values have remained stable between six months and two years of treatment) warrants further investigation.

### In patients with the attenuated phenotype of MPS II, the evaluation of HS levels in the CSF should be carried out before the start of treatment and at six months of treatment. After two years of treatment, either the quantification of HS in CSF or neurocognitive assessment can be performed

Although patients with the attenuated phenotype of MPS II generally present little or no cognitive impairment, the authors consider the reduction of HS in CSF a relevant indicator of disease improvement, regardless of severity, because the accumulation of GAGs represents the primary pathophysiological change in MPS. By contrast, in individuals without MPS, HS concentrations in CSF are very low or undetectable [12].

Therefore, the authors recommend that patients with the attenuated phenotype of MPS II undergoing treatment with any drug that crosses the blood-brain barrier should have their CSF levels of HS evaluated using the same scheme defined for the severe form. To reach a consensus on this issue, the authors considered the same factors described in the previous topic.

For research purposes, it would be ideal to maintain the same protocol to allow comparison of results from both severe and attenuated phenotype cases. However, in the real world, it might prove more difficult for the patient’s family to accept the collection of CSF, if the patient presents an attenuated phenotype. In addition, patients may face greater difficulties accessing the procedure. The understanding of the expert group is that patients with attenuated disease have lower CNS levels of HS, compared to patients with severe disease, and should not be submitted to regular lumbar punctures after two years of treatment, as no major modifications on the levels are expected. Thus, the experts agreed that, after two years of treatment, the physician can decide between quantifying HS in CSF and/or conducting neurocognitive evaluations.

The panel emphasized that neurocognitive evaluations should be carried out by an experienced professional and include the application of psychometric tests. Moreover, it is essential to perform a baseline assessment before treatment, so the patient can serve as their own reference (e.g. if they have Attention Deficit Hyperactivity Disorder - ADHD or autism, this will not interfere with subsequent evaluation).

### There is an association between reduction of HS levels in CSF and clinical improvement of signs and symptoms in patients with MPS II. However, it is not possible to establish a direct correlation between HS values and specific clinical outcomes

There was a consensus among the authors that there is a clear association between reduction of HS levels in CSF and improvement of signs and symptoms in MPS II patients - including non-neurological cases - as demonstrated in clinical studies with intracerebroventricular enzyme replacement therapy and blood-brain barrier-crossing drugs [11, 13, 15, 19], and by observations of patients in daily practice by specialists. Nevertheless, to date, no direct correlation between specific levels of HS in CSF and type of clinical manifestation has been identified and no specific clinical outcome expected according to a given reduction in HS after treatment has been observed.

It should be acknowledged that in patients who already have important neurological damage, it is not expected that measurable clinical benefits will occur even with decrease of the CSF levels of heparan sulfate, as indicated in the trial described by Muenzer et al., 2022 [18].

## Conclusions

The authors consider HS in CSF a valuable biomarker for assessing neurological severity in patients with MPS II, as well as for monitoring the treatment of these patients with drugs that cross the blood-brain barrier, despite the challenges associated with the collection and analysis of CSF. However, it is essential to ensure that the method of HS quantification employed is consistent, so that the interpretation of results enables accurate differentiation between patients with severe and attenuated phenotypes, according to the established cut-off value.

It is essential to consider the impact of treatment interruptions and patient adherence when analyzing real-world outcomes, and also to consider these factors to define the most appropriate timing of CSF collection for therapeutic monitoring.

In MPS II patients with the attenuated phenotype, the experts recommended that, after two years of treatment, HS measurement in CSF can be replaced by neurocognitive evaluations in cases when collecting and/or analyzing the CSF proves difficult. However, the panel emphasized that these tests must be conducted by experienced and trained professionals.

Despite all the evidence discussed in this article, currently it is not possible to correlate CSF HS levels with specific clinical manifestations and/or outcomes. In addition, it will be important to consider the level of CNS damage already in place, as for more advanced cases the impact of decreasing CSF HS levels may be lower than for cases treated earlier in the course of the disease. Future studies should provide relevant information to understand this relationship, as well as the presence or absence of correlation between HS levels in CSF versus serum, plasma, and urine, the transport mechanism of HS from the brain to the CSF, and of the role of elevated HS levels in the pathophysiology of neurological disease in MPS II.

## Data Availability

Not applicable.

## Abbreviations

CNS: Central Nervous System
CSF: Cerebrospinal Fluid
GAGs: Glycosaminoglycans
HS: Heparan Sulfate
IDS: Iduronate-2-Sulfatase Gene
MPS II: Mucopolysaccharidosis Type II
MRI: Magnetic Resonance Imaging
